# "Ierne" at Fault

**Published:** 1919-11-22

**Authors:** 


### "IERNE" AT FAULT.

AT the end of "Ierne's" article published on page 146 of our last issue the statement is made that the nurse is being driven into trade unionism, for "she has paid her subscriptions to many societies but none has so far lifted a finger to minister to her pressing necessities." We are sure "Ierne" will regret having done grave injustice to the College, being apparently obsessed by anger at the sparse measure extended to nurses by the Stage Army group of societies to which nurses have paid their subscriptions.

Sir Arthur Stanley's letter effectually demonstrates, however, that "Ierne's" condemnation does not apply, and is not in fact true, but the precise opposite, when applied to the College of Nursing. Unfortunately our readers have not only believed that "Ierne" referred to the College, but meant to include it. A zealous College supporter writes: "I really must protest against some of the statements in 'Ierne's' article in THE HOSPITAL of November 15. For instance, in the last sentence: 'She has paid her subscriptions to many societies but none has so far lifted a finger to minister to her pressing necessities.' Now this is absolutely untrue. I have had evidence in several cases of the fact that hospitals did increase their nurses' pay owing to the College Report. And through the Nation's Fund over eighty cases of distressed nurses are being treated every week. Articles and untrue statements such as 'Ierne's,' especially when appearing in a paper otherwise so friendly to the College, must tend to drive the nurses to trade unionism." We hope, in view of the facts set forth in Sir Arthur Stanley's letter above, and what our correspondent states, that the exact contrary will be the case, for this publication of the want of foundation for "Ierne's" statement, so far as the College of Nursing is concerned, must tend to convince nurses that the right course for them to take is to support the College and increase the membership by every means in their power between now and the annual meeting next spring. We hope before Christmas that every reader of THE HOSPITAL who is a member of the College will have secured at least one recruit, so as to bring the total membership up to at least 20,000 trained nurses.

